# Ten new *ATM* alterations in Polish patients with ataxia-telangiectasia

**DOI:** 10.1002/mgg3.98

**Published:** 2014-07-30

**Authors:** Marta Joanna Podralska, Agnieszka Stembalska, Ryszard Ślęzak, Aleksandra Lewandowicz-Uszyńska, Barbara Pietrucha, Sylwia Kołtan, Jadwiga Wigowska-Sowińska, Jacek Pilch, Maria Mosor, Iwona Ziółkowska-Suchanek, Agnieszka Dzikiewicz-Krawczyk, Ryszard Słomski

**Affiliations:** 1 Institute of Human Genetics of the Polish Academy of SciencesPoznań, Poland; 2 Department of Genetics, Wroclaw Medical UniversityWroclaw, Poland; 3 3rd Department and Clinic of Paediatrics, Immunology and Rheumatology of Developmental Age, Wroclaw Medical UniversityWroclaw, Poland; 4 Department of Immunology, The Children's Memorial Health InstituteWarsaw, Poland; 5 Department of Pediatrics, Hematology and Oncology, Institute of Pediatrics, Medical AcademyBydgoszcz, Poland; 6 Department of Developmental Neurology, University of Medical SciencesPoznań, Poland; 7 Department of Child Neurology, Medical University of SilesiaKatowice, Poland

**Keywords:** Ataxia telangiectasia, * ATM *, Polish population, mutation analysis, sequencing, MLPA

## Abstract

Inherited biallelic mutations of the *ATM* gene are responsible for the development of ataxia telangiectasia (AT). The objective of the present study was to conduct molecular analysis of the *ATM* gene in a cohort of 24 Polish patients with ataxia-telangiectasia with aim being to provide an updated mutational spectrum in Polish AT patients. As a result of molecular analysis, the status of recurrent mutation was confirmed and ten new ATM variants were detected. Application of MLPA analysis allowed the detection of large genomic deletion. Previously, this type of mutation had never been seen in our population. Finally, in silico analysis was carried out for newly detected ATM alterations. In addition, functional analysis was performed to evaluate the effects of intronic variants: c.3402+30_3402+32delATC.

## Introduction

Ataxia-telangiectasia (AT, MIM#208900) is a neurodegenerative disorder belonging to primary immunodeficiency diseases and it is associated with DNA repair defects. AT is inherited in an autosomal recessive manner and results from mutations in the ataxia telangiectasia mutated gene (*ATM*, MIM*607585). Protein encoded by *ATM* plays an important role in monitoring and maintaining DNA integrity. ATM coordinates cell cycle progression and the cellular response to DNA double-stranded breaks by phosphorylation of several substrates: TP53, BRCA1, CHEK2, and nibrin (Derheimer and Kastan 2010; Keimling et al. 2011). The first symptoms of ataxia-telangiectasia often appear in early childhood, when children begin to walk. The most characteristic manifestations of AT are neurological dysfunction (ataxia) and dilated blood vessels (telangiectasia) in corner of the eyes and in the skin on the ears and cheeks. Neurological manifestations presented by AT patients resulted from cerebellar atrophy (Carlessi et al. 2013). Neurodegeneration of the cerebellum is progressive and is responsible for unsteady gait, poor muscle control, abnormal eye movements and problems with speaking or swallowing (McKinnon 2004). Immunodeficiency is presented by more than half of all patients with AT. Furthermore, the humoral immune system, cellular immune system or both can be affected. Immunoglobulin levels (particularly IgA, IgE, and IgG2) are diminished or absent. Common abnormality of cell-mediated immunity is peripheral lyphmopenia, and especially CD4 T-cells are reduced. Patients with AT suffer from sinopulmonary infections, but opportunistic infections are rare (Lumsden et al. 2004; Staples et al. 2008). One of the biomarkers of AT is an elevated *α*-fetoprotein (AFP) level in serum. In cytogenetic studies the translocation between 7 and 14 chromosomes is identified in 5–15% cases. Patients with AT have a strong predisposition to malignancy, with an increased risk of leukemia and lymphoma of both B-cell and T-cell origins. In AT patients, the most frequent malignancies are found in the lymphoid system, and T-cell tumors occur more frequently than B-cell tumors. Patients living longer also present with other type of cancers, like ovarian and breast cancer, gastric cancer, melanoma, leiomyomas, and sarcomas (Byrd et al. 1996; Reiman et al. 2011).

## Materials and Methods

### Patients

26 AT patients from 24 unrelated families were recruited from the department of Immunology and Genetics departments in Poland. The majority of our patients presented typical ataxia-telangiectasia manifestation: immunoglobulin deficiencies involving: IgA, IgE, and IgG, and high levels of alfa-fetoprotein (*AFP*). Cerebellar ataxia causing uncoordinated movement, swallowing difficulties and dysarthria were observed in our patients. Telangiectasia were detected in only part of the AT patient group. The clinical features of the individual A-T patients were summarized in Table1.

**Table 1:** Clinical manifestations, laboratory findings of AT patients

Patients	Age	Sex	Ataxia (age)	Telangiectasia (age)	Afp	Immunoglobulins
AT01	11	M	−	−		↓IgG
AT02	7	F	+(1.8)	−		↓IgA
AT06	7	M	+	+	↑	↓IgA, ↓IgG, ↑IgM
AT07	5	F	+(1.8)	−	↑	↓IgA, ↓IgG
AT7.1	5	M	+(1.8)	−	↑	↓IgA, ↓IgG
AT08	6	F	+	+	↑	↓IgA, ↓IgG2
AT10	14	M	+(1.3)	+(9,5)	↑	↓IgA, ↓IgG2
AT12	3	M	+(1.4)	+(3)		↓IgG3
AT13	6	F	+(1.4)	+(3)	↑	↓IgA, ↓IgG2
AT15	17	M	+(1.5)	+(7)	↑	↓IgA
AT19	2	M	+(1.2)	−	↑	↓IgG3
AT21	9	F	+		↑	↓IgA
AT23	4	M	+(2.1)	+	↑	↓IgA, ↓IgG
AT24	16	M	+	+	↑	↓IgA
AT26	21	F	+		Norm	Norm
AT27	4	M	+(1.8)	−	↑	↓IgA
AT28	9	M	+	+	↑	↓IgA
AT30	9	F	+(2)	+(6)	↑	↓IgA, ↓IgG2, ↓IgG4
AT31	13	M	+(1.3)	+(2)	↑	↓IgA, ↓IgG2
AT33	3	M	+(1)	+(2)	↑	↓IgA
AT33.1	3	M	+(1)	+(2)	↑	↓IgA
AT34	5	F	+(2)	−	Norm	↓IgA,↓IgG2, ↓IgG3, ↓IgG4
AT35	12	M	+(1.5)	+(8)	↑	↓IgA, ↓IgG2,
AT36	4	M	+(1)	−	↑	↓IgA, ↓IgG2, ↓IgG3, ↓IgG4
AT37	8	F	+	+	↑	↑IgG
AT38	5	M	+	+	↑	↓IgG

F, female; M, male; +, present; –, absent; arrows indicate increase (pointing up)/decrease (pointing down) level of an AFP/immunoglobulin.

### Molecular analysis

We performed genomic DNA extraction from peripheral ethylenediamine tetraacetic acid-anticoagulated blood samples using standard phenol-chloroform protocols. The genomic DNA was amplified using a previously reported primers set, flanking all exons and exon/intron boundaries of the *ATM* gene (Castellvi-Bel et al. 1999). Single-strand conformation polymorphism (*SSCP*) and heteroduplex (*HD*) were performed and the products were visualized with silver staining. PCR products with variant migration patterns were sequenced. Multiplex Ligation-dependent Probe Amplification (*MLPA*) was performed with P041 and P042 kits (MRC Holland, Amsterdam, The Netherlands) in accordance with the manufacturer's instructions. MLPA products were analyzed on an ABI sequencer. Data analysis was performed by exporting the peak areas to a Microsoft Excel file.

We investigated the effect of a c.3402+30_3402+32delATC intronic variant on splicing and expression. Total RNA was obtained from the lymphoblastoid cell line (LCLs). RNA was isolated from two patients carrying c.3402+30_3402+32delATC and from controls by using a *QIAmp RNA blood mini kit* (Qiagen, Hilden, Germany) and was reverse-transcribed into cDNA using *Superscript III Reverse Transcriptase* (Invitrogen, Carlsbad, CA). Expression of the *ATM* gene was measured quantitatively by real-time PCR using *KAPA SYBR*^*®*^ *FAST One-Step qRT-PCR Kits* (KAPA Biosystem, Boston, MA) by gene specific primers and *β*-actin was used as a reference control. The immunoblotting analysis was performed using an ATM 2C1 monoclonal antibody raised against amino acids 2577-3056 and an antibody against *β*-actin as an internal loading control (both antibodies; Santa Cruz Biotechnology, Inc., Heidelberg, Germany).

In silico analyses of the *ATM* variants were performed using the Protein Variation Effect Analyzer (PROVEAN, J. Craig Venter Institute), Align Grantham Variation Grantham Deviation (Align GVGD, International Agency for Research on Cancer, Lyon, France) and Alibaba 2.1 TF Binding Prediction (BIOBASE), which are freely available web-based programs.

The reference sequence for *ATM* used GenBank NM_000051.3. Mutation numbering uses the A of the ATG initiation codon as +1.

The study was conducted with the approval by the Central Ethical Committee of the Ministry of Health, Poland, in accordance with the tenets of the Declaration of Helsinki.

## Results and Discussion

The screening of the *ATM* gene in 24 AT families revealed 38 changes in the DNA sequence. The rate of DNA alterations in this series of AT patients is approximately 80% (38 detected variants/48 expected mutations). The mutation types are diverse, including 21 nonsense (55.3%), 12 splicing (31.6%), 3 large genomic deletions (7.9%) and 2 missense alterations (5.3%). All *ATM* changes and further details are shown in Table2. The majority of AT patients were compound heterozygotes. Only two patients out of 24 were found to be homozygous (AT24 [c.4007_4008insA; c.4007_4008insA], AT37 [c.9021_9022insA; c.9021_9022insA]). As published previously, few recurring mutations were detected in Polish AT patients (c.5932G>T, c.6095G>A, c.7630-2A>C, c.7010_7011delGT) (Telatar et al. 1998; Mitui et al. 2005; Demuth et al. 2011). The c.6095G>A and c.7630-2A>C are splicing mutations causing exon skipping, 43 and 53, respectively. The most frequent mutations among our AT patients are: c.6095G>A (5 times), c.7630-2A>C (6), c.5932G>T (6), c.7010_7011delGT (2). Recurrent mutations cover 76.3% (29/38) of all detected mutations. Several families in the Polish population had newly diagnosed DNA alterations (10/38; 21.05%). Ten changes in the *ATM* gene were novel: c.8441delC, c.6145T>G, c.434T>G, c.6754_6754delA, c.4007_4008insA, c.7606G>A, c.3402+30_3402+32delATC, deletion of exons 19-20, deletion of exon 63, deletion of exons 62 and 63 of *ATM* gene). Seven newly discovered *ATM* alterations resulted in the exchange of the amino acid into a stop codon or are products of a frameshift error generating the stop codon and truncating the protein product. The remaining new alterations are substitutions and one intronic variant. We identified the replacement of non-polar, hydrophobic leucine by basic amino arginine at position 145 and the substitution of aromatic tyrosine for acidic amino aspartic acid at position 2049 in the protein sequence. To predict the biological effect of two missense changes, in silico analysis was performed using the Protein Variation Effect Analyzer (PROVEAN, J. Craig Venter Institute). The algorithm of analysis predicted c.434T>G (score −3.403) and c.6145T>G (score −6.956) to be pathogenic. A multiple sequence alignment was made with the Align Grantham Variation Grantham Deviation program (Align GVGD, International Agency for Research on Cancer, Lyon, France), and is presented in Table3.

**Table 2:** *ATM* mutations of 24 families with AT

Patients	DNA level	Protein level	Consequence	Status	Genotype
AT01	c.8441delG	p.Glu2814LysfsTer43	Truncation	Novel	Compound heterozygote
	c.6095G>A	Exon 43 skipped	Aberrant splicing	Li and Swift (2000); Mitui et al. (2005); Sandoval et al. (1999); Telatar et al. (1998)	
AT02	c.3402+30_3402+32delATC	?	?	Novel	Compound heterozygote
AT02.1	c.3402+30_3402+32delATC	?	?	Novel	Carrier
AT02.2	Excluded c.3402+30_3402+32delATC				
AT06	c.6145T>G	p.Tyr2049Asp	Missense	Novel	Compound heterozygote
	c.434T>G,	p.Leu145Arg	Missense	Novel	
AT07	c.6754_6754delA	p.Thr2252ProfsTer5	Truncation	Novel	Compound heterozygote
	c.6095G>A	Exon 43 skipped	Aberrant splicing	Li and Swift (2000); Mitui et al. (2005); Sandoval et al. (1999); Telatar et al. (1998)	
AT7.1	c.6754_6754delA	p.Thr2252ProfsTer5	Truncation	Novel	Compound heterozygote
	c.6095G>A,	Exon 43 skipped	Aberrant splicing	Li and Swift (2000); Mitui et al. (2005); Sandoval et al. (1999); Telatar et al. (1998)	
AT08	c.7630-2A>C	Exon 54 skipped	Aberrant splicing	Li and Swift (2000); Mitui et al. (2005); Sandoval et al. (1999); Telatar et al. (1998)	Compound heterozygote
AT08.1	c.7630-2A>C	Exon 54 skipped	Aberrant splicing	Li and Swift (2000); Mitui et al. (2005); Sandoval et al. (1999); Telatar et al. (1998)	Carrier
AT10	c.6095G>A,	Exon 43 skipped	Aberrant splicing	Li and Swift (2000); Mitui et al. (2005); Sandoval et al. (1999); Telatar et al. (1998)	Compound heterozygote
AT12	c. 7630-2A>C	Exon 54 skipped	Aberrant splicing	Li and Swift (2000); Mitui et al. (2005); Sandoval et al. (1999); Telatar et al. (1998)	Compound heterozygote
AT13	Deletion of 62 and 63 exons		Truncation	Novel	Compound heterozygote
	c.5932G>T	p.Glu1978Ter	Truncation	Birrell et al. (2005); Li and Swift (2000); Mitui et al. (2005)	
AT15	c.1179_1180delGG	p.Trp393Ter	Truncation	Buzin et al. (2003)	Compound heterozygote
AT19	c.6095G>A	Exon 43 skipped	Aberrant splicing	Li and Swift (2000); Mitui et al. (2005); Sandoval et al. (1999); Telatar et al. (1998)	Compound heterozygote
AT21	c.7010_7011delGT			Mitui et al. (2005); Telatar et al. (1996)	Compound heterozygote
	c.5932G>T	p.Glu1978Ter	Truncation	Birrell et al. (2005); Li and Swift (2000); Mitui et al. (2005)	
AT23	c.381_381delA	p.Thr127ThrfsTer2	Truncation	Babaei et al. (2005); Castellvi-Bel et al. (1999); Mitui et al. (2005)	Compound heterozygote
	c.3402+30_3402+32delATC	?	?	Novel	
AT23.1	Excluded c.381_381delA				Carrier
	c.3402+30_3402+32delATC	?	?	Novel	
AT23.2	c.381_381delA	p. Thr127Thr fsTer2	Truncation	Babaei et al. (2005); Castellvi-Bel et al. (1999) Mitui et al. (2005)	Carrier
	Excluded c.3402+30_3402+32delATC				
AT24	c.4007_4008insA	p.Phe1336PhefsTer3	Truncation	Novel	Homozygote
	c.4007_4008insA	p.Phe1336PhefsTer3	Truncation	Novel	
AT24.1	c.4007_4008insA	p.Phe1336PhefsTer3	Truncation	Novel	
AT26	c.7606G>A	p.Gly2536Ter	Truncation	Novel	Compound heterozygote
AT27	c.3402+30_3402+32delATC	?	?	Novel	Compound heterozygote
	c.5932G>T	p.Glu1978Ter	Truncation	Birrell et al. (2005); Mitui et al. (2005)	
AT27.1	c.5932G>T	p.Glu1978Ter	Truncation	Birrell et al. (2005); Li and Swift (2000); Mitui et al. (2005)	Carrier
AT27.2	c.3402+30_3402+32delATC	?	?	Novel	Carrier
AT28	c.5932G>T	p.Glu1978Ter	Truncation	Birrell et al. (2005); Li and Swift (2000); Mitui et al. (2005)	Compound heterozygote
	c.7630-2A>C	Exon 54 skipped	Aberrant splicing	Li and Swift (2000); Mitui et al. (2005); Sandoval et al. (1999); Telatar et al. (1998)	
AT28.1	c.5932G>T	p.Glu1978Ter	Truncation	Birrell et al. (2005); Li and Swift (2000); Mitui et al. (2005)	Carrier
	Excluded c.7630-2A>C				
AT30	c.6095G>A	Exon 43 skipped	Aberrant splicing	Li and Swift (2000); Mitui et al. (2005); Sandoval et al. (1999); Telatar et al. (1998)	Compound Heterozygote
AT31	c.2250G>A		Aberrant splicing	Byrd et al. (1996); Mitui et al. (2003); Sandoval et al. (1999)	Compound heterozygote
	c.7630-2A>C	Exon 54 skipped	Aberrant splicing	Li and Swift (2000); Mitui et al. (2005); Sandoval et al. (1999); Telatar et al. (1998)	
AT33	c.7630-2A>C	Exon 54 skipped	Aberrant splicing	Li and Swift (2000); Mitui et al. (2005); Sandoval et al. (1999); Telatar et al. (1998)	Compound heterozygote
	c.5932G>T	p.Glu1978Ter	Truncation	Birrell et al. (2005); Li and Swift (2000); Mitui et al. (2005)	
AT33.1	c.7630-2A>C	Exon 54 skipped	Aberrant splicing	Li and Swift (2000); Mitui et al. (2005); Sandoval et al. (1999); Telatar et al. (1998)	Compound heterozygote
	c.5932G>T	p.Glu1978Ter	Truncation	Birrell et al. (2005); Li and Swift (2000); Mitui et al. (2005)	
AT34	Deletion of exons 19 and 20		Truncation	Novel	Compound heterozygote
	Deletion of exon 63		Truncation	Novel	
AT35	c.5932G>T	p.Glu1978Ter	Truncation	Birrell et al. (2005); Li and Swift (2000); Mitui et al. (2005)	Compound heterozygote
AT36	c.3802_3802delG	p.Val1268Ter	Truncation	Mitui et al. (2003); Sandoval et al. (1999)	Compound heterozygote
AT37	c.9021_9022insA	p.Arg3008ThrfsTer54	Truncation	Mitui et al. (2005)	Homozygote
	c.9021_9022insA	p.Arg3008ThrfsTer54	Truncation		
AT37.1	c.9021_9022insA	p.Arg3008ThrfsTer54	Truncation	Mitui et al. (2005)	Carrier
AT37.2	c.9021_9022insA	p.Arg3008ThrfsTer54	Truncation	Mitui et al. (2005)	Carrier
AT38	c.7010_7011delGT	p.Gly2337SerfsTer35	Truncation	Telatar et al. (1996)	Compound heterozygote
	c.7630-2A>C	Exon 54 skipped	Aberrant splicing	Li and Swift (2000); Mitui et al. (2005); Sandoval et al. (1999); Telatar et al. (1998)	
AT38.1	Excluded c.7010_7011delGT				

On the basis of transcripts NM_000051 for *ATM*.

**Table 3:**
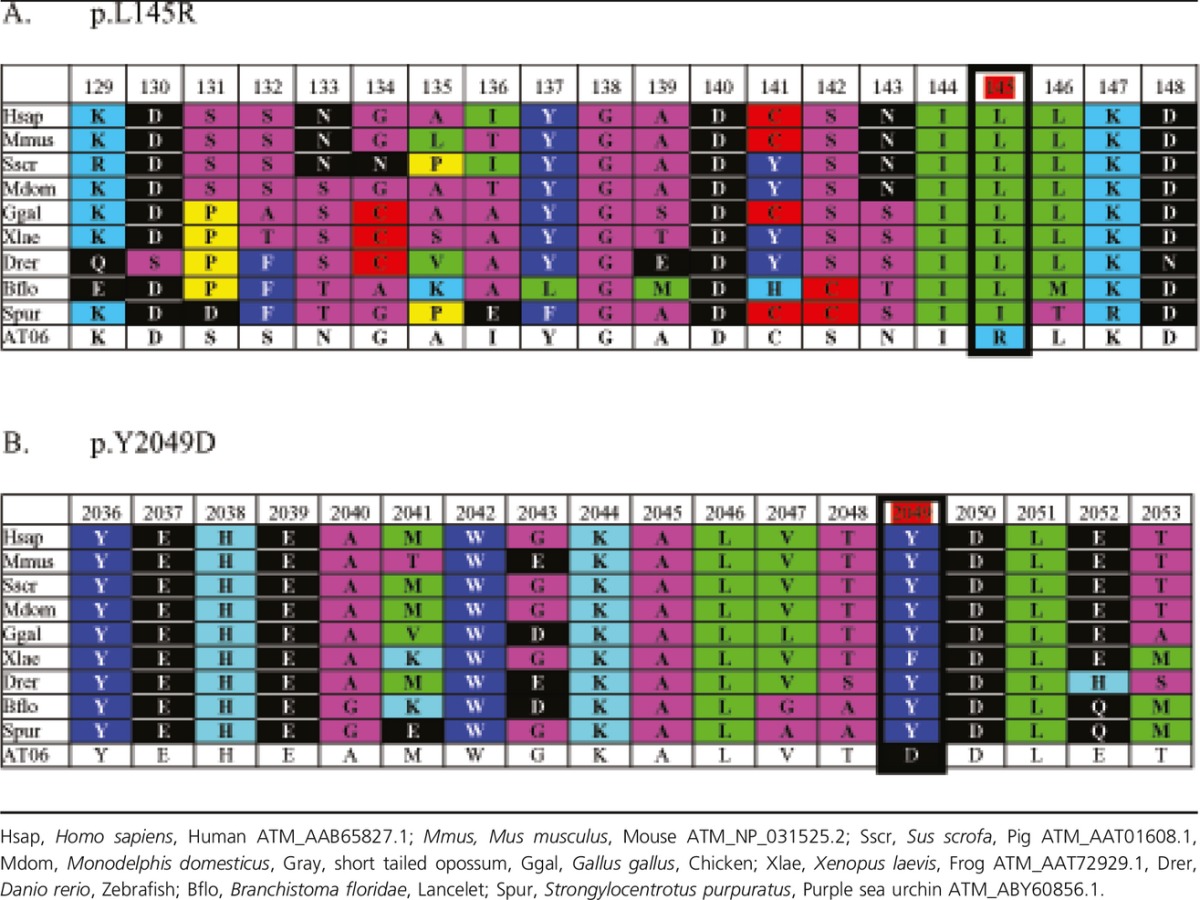
Multiple alignment of regions surrounding L145 (A) and Y2049 (B) of ATM across different organisms

**Figure 1:**
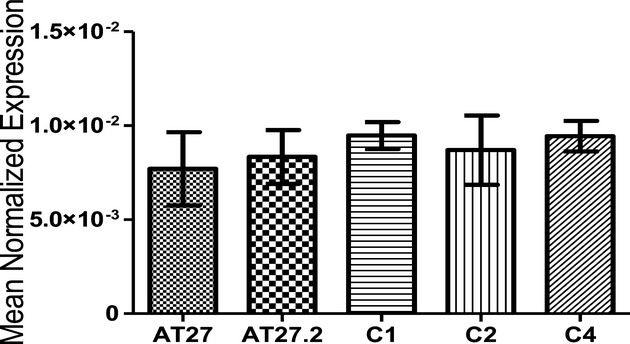
Real-time PCR results for ATM mRNA levels. ATM mRNA levels were measured by RT-PCR from controls and individuals with c.3402+30_3402+32delATC and normalized to β-actin mRNA levels. Data are expressed as mean normalized expression ± s.d. The one-way ANOVA followed by Newman-Keuls test was performed to determine the significance. There are no significant differences in expression between patients with c.3402+30_3402+32delATC and controls.

The other new change in the *ATM* sequence in the Polish population was c.3402+30_3402+32delATC in intron 25. Splicing defects in the *ATM* gene are common (Teraoka et al. 1999). Most of these involve disruption of the canonical splice sites and lead to exon skipping. Furthermore, deep intronic mutation was described previously (Sutton et al. 2004; Coutinho et al. 2005). For example, in the United Kingdom, 15% of AT families are intronic c.5762-1050A>G mutation carriers. This mutation activates a cryptic splice donor/acceptor site, resulting in the insertion of 137 nucleotides of an intronic sequence (McConville et al. 1996). The c.3402+30_3402+32delATC was identified in three of our AT families (3/24, 12.5%). This intronic variant appeared in a heterozygous state in all cases. Among these 200 controls, this intronic variant was not observed. According to data from the NHLBI Exome Sequencing Project, the ATC deletion allele has a frequency of 0.11% (14/12504) of total alleles studied and is not observed in a homozygous state. We subsequently analyzed the *ATM* transcripts, to investigate the possible effects of intronic deletions. No abnormal *ATM* transcripts were detected. Moreover, in silico analysis showed that an intronic variant may damage the transcription factor (TF) binding sites, resulting in the disruption of the Oct-1 and GATA1 binding sites and the appearance of the binding site for other TF and Pit1 sites. Up to now, there has been no report of immediate interactions between these three TF proteins and the *ATM* gene. Oct-1 and Pit1 belong to a large POU family of transcription factors. Oct-1 is known as a transcription factor involved in regulation of some housekeeping genes, histone H2B, snRNAs as well as in tissue-specific regulation of immunoglobulin and mediated antigen-independent B cell development. GATA1 is implicated in the reprograming of hematopoietic precursors and the regulation of G_1_/S cell cycle progression. Also it is known that three TFs bind to intronic regions and affect the gene expression. On the basis of these data, *ATM* mRNA was measured by quantitative real-time PCR with *β*-actin as an internal reference gene. The results showed that the mRNA level in the samples with c.3402+30_3402+32delATC is similar to the mRNA level in control cases (Fig. 1). However, the second allele without intronic variant can be up-regulated to compensate for the lack of a function of the defective allele. On the other hand, the *ATM* tissue-specific expression depending on Oct, Pit-1 or GATA1 is also not excluded. A total loss of the ATM protein was detected by western blotting in patients carrying this intronic variant. This observation supports the hypothesis that second allele can be up-regulated. In spite of the initial results, other functional analysis may reveal that the c.3402+30_3402+32delATC is a pathogenic mutation.

In patient AT13, the large genomic deletion of exons 62 and 63 was detected. This deletion is combined with a nonsense mutation c.5932G>T in exon 42 (Table3). Another recent interesting case is a patient with two large deletions. The first deletion encompasses two exons 19–20 and is combined with a deletion removing the last exon of *ATM*. Previous reports estimated that the large genomics mutations in *ATM* are detected in 2% to 23% of AT patients. A high percentage of large genomic mutations was described in the Japanese population. There have been a few reports showing that large genomic deletion (LGD) occurs in Brazilian, Chinese, Costa Rican, Dutch, and Japanese ataxia telangiectasia patients (Broeks et al. 1998; Coutinho et al. 2004; Nakamura et al. 2012; Huang et al. 2013). Moreover, Cavalieri et al. reported a large duplication in the *ATM* gene, spanning exons 4–20 (41kbp) (Cavalieri et al. 2008). LGDs were localized in a different part of the *ATM* gene, especially in the last two exons. Previous analyses of the genomic deletions of last two exons of the *ATM* gene show that mutations are caused by retro-transposable elements (long interspersed element-1, LINE1). The 3′ end and downstream sequence of the *ATM* gene are riddled with retrotransposons (ALU, LINE).

In summary, in this study, we confirmed the status of recurrent mutations (c.5932G>T, c.6095G>A, c.7630-2A>C) and also detected ten new *ATM* gene changes in Polish patients with AT. In the future, further investigations on the functional role and clinical impact of novel alterations will be performed.
